# Novel Pedicle Screw and Plate System Provides Superior Stability in Unilateral Fixation for Minimally Invasive Transforaminal Lumbar Interbody Fusion: An In Vitro Biomechanical Study

**DOI:** 10.1371/journal.pone.0123134

**Published:** 2015-03-25

**Authors:** Jie Li, Hong Xiao, Qingan Zhu, Yue Zhou, Changqing Li, Huan Liu, Zhiping Huang, Jin Shang

**Affiliations:** 1 Department of Orthopedics, Xinqiao Hospital, The Third Military Medical University, Chongqing, China; 2 Department of Orthopaedic and Spinal Surgery, Nanfang Hospital Southern Medical University, Guangzhou, Guangdong, China; University of Michigan, UNITED STATES

## Abstract

**Purpose:**

This study aims to compare the biomechanical properties of the novel pedicle screw and plate system with the traditional rod system in asymmetrical posterior stabilization for minimally invasive transforaminal lumbar interbody fusion (MI-TLIF). We compared the immediate stabilizing effects of fusion segment and the strain distribution on the vertebral body.

**Methods:**

Seven fresh calf lumbar spines (L3-L6) were tested. Flexion/extension, lateral bending, and axial rotation were induced by pure moments of ± 5.0 Nm and the range of motion (ROM) was recorded. Strain gauges were instrumented at L4 and L5 vertebral body to record the strain distribution under flexion and lateral bending (LB). After intact kinematic analysis, a right sided TLIF was performed at L4-L5. Then each specimen was tested for the following constructs: unilateral pedicle screw and rod (UR); unilateral pedicle screw and plate (UP); UR and transfacet pedicle screw (TFS); UP and TFS; UP and UR.

**Results:**

All instrumented constructs significantly reduced ROM in all motion compared with the intact specimen, except the UR construct in axial rotation. Unilateral fixation (UR or UP) reduced ROM less compared with the bilateral fixation (UP/UR+TFS, UP+UR). The plate system resulted in more reduction in ROM compared with the rod system, especially in axial rotation. UP construct provided more stability in axial rotation compared with UR construct. The strain distribution on the left and right side of L4 vertebral body was significantly different from UR and UR+TFS construct under flexion motion. The strain distribution on L4 vertebral body was significantly influenced by different fixation constructs.

**Conclusions:**

The novel plate could provide sufficient segmental stability in axial rotation. The UR construct exhibits weak stability and asymmetrical strain distribution in fusion segment, while the UP construct is a good alternative choice for unilateral posterior fixation of MI-TLIF.

## Introduction

Generally, posterior pedicle screw and rod construct fixation after minimally invasive transforaminal lumbar interbody fusion (MI-TLIF) is frequently used to provide spinal stability until the segment accomplishes solid fusion [1–3]. Unilateral pedicle screw and rod construct is gaining acceptance for less operation time and a shorter length of hospital stay [4–6]. Although the extent of stability required for spinal fusion is unknown, unilateral pedicle screw constructs have been reported to result in inadequate spine stiffness with inferior results in MI-TLIF [7]. Unilateral pedicle screws fixation cannot provide sufficient stability in lateral bending and axial rotation, and pedicle screws endure more stress in biomechanical studies [8–10]. A new posterior fixation construct should be designed to provide more stability in unilateral fixation mode.

The pedicle screw and plate systems have been historically used routinely, but preferences gradually shifted to pedicle screw and rod systems [11]. The structural imperfection and the inappropriate use in multi-segmental posterolateral fusion caused the dissatisfying clinical results. The fixed screw-hole distances and the lack of an accommodation angle between the screw and longitudinal element were the main structure limitation of the old plate systems [11, 12], which made it difficult to set the pedicle screw or plate. Additionally, less rigid plate could not bear load transduction in multi-segmental posterolateral lumbar fusion [11]. The inferior results with the old plate system included high rate of pseudarthrosis and hardware failure [13–15], and lower fusion rate compared with the rod system [16]. However, lumbar fusion surgery has recently shifted from posterolateral fusion to interbody fusion, and also shifted from long segment fusion to short segment fusion. The concept of minimally invasive technique and less implant profile have been advocated by more surgeons [11]. The development of interbody fusion techniques changed the role of posterior instrumentation, from principal load-bearing to tension band and neutralization [9]. MI-TLIF has become the prevalent technique for lumbar degenerative diseases, and more new instrumentation methods have been reported by clinical or biomechanical studies [8, 9]. If a new pedicle screw and plate system overcame all the structural defects of the old one and got feasible use in minimally invasive surgery, would it be used again?

**Fig 1:**
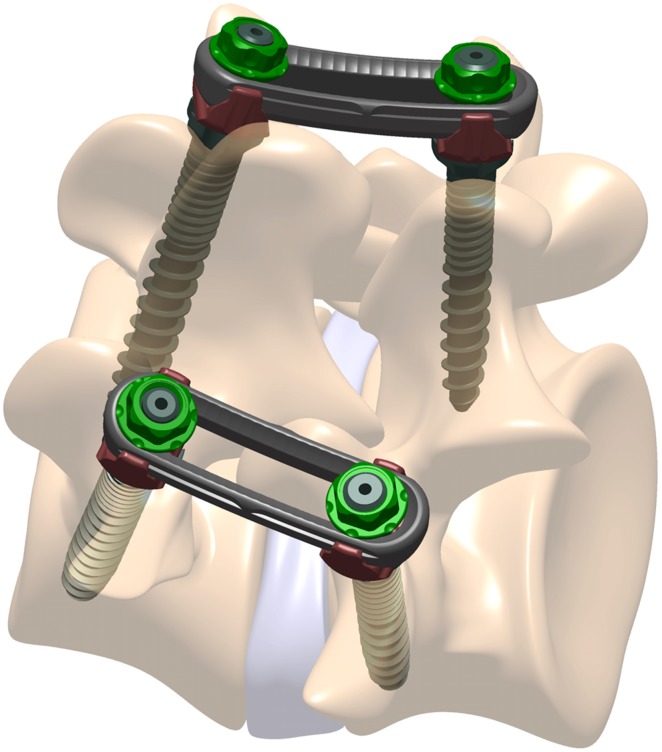
The pedicle screw and plate system schematic diagram.

**Fig 2:**
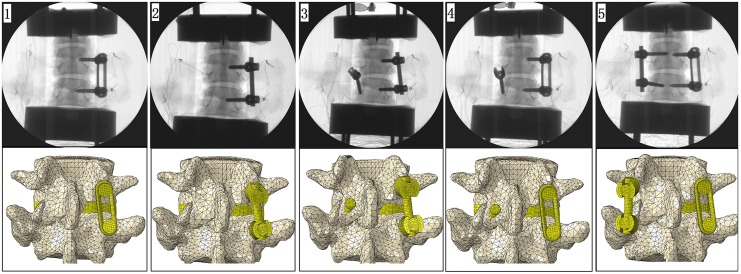
Surgical constructs: unilateral pedicle screw and plate (UP); unilateral pedicle screw and rod (UR); unilateral pedicle screw and rod + transfacet pedicle screw (UR+TFS); unilateral pedicle screw and plate + transfacet pedicle screw (UP+TFS); unilateral pedicle screw and plate + unilateral pedicle screw and rod (UP+UR). Some part of the facet screw and the plate constructs were ignored in the finite element figures.

In the present biomechanical study, the authors describe a novel pedicle screw and plate system for one-level MI-TLIF (Fig 1). Monoaxial pedicle screws are used in the new system to facilitate the distraction-compression function of MI-TLIF. The spherical surface slider on plate controls the screw and plate interface angle, functionally converting it to a polyaxial construct and allowing 15° of freedom in plate setting. The plate construct could provide more stability in axial rotation in theory, which enhances the fixation effect of unilateral pedicle screw fixation. The purpose of this study is to evaluate the stability of a spinal motion segment in TLIF model with the novel plate system. This system is compared with traditional pedicle screw and rod system by different asymmetrical posterior stabilization methods (Fig 2).

## Materials and Methods

### Specimen preparation

Seven fresh calf specimens (range, 6–8 weeks old), consisting of L3–L6, were used in this study. The specimens were obtained from Yun-Jing food market in Guangzhou and the study was approved by the Ethics Committee of the Second Affiliated Hospital of Third Military Medical University. After examination of the specimen, plain radiographs were taken to exclude fracture and deformities. Specimens were obtained fresh-frozen at −20°C and then thawed in a bath of normal saline at 30°C before experiment. The average time between slaughter and experimentation was 7 days. The paravertebral musculature was removed, while all ligaments, joints and disc spaces were preserved. Short screws were partially driven into the L3 and L6 vertebrae to obtain a better anchorage of the vertebrae in the plaster powder. Then, the L3 and L6 vertebrae were embedded in plaster. Plexiglas markers, each with four infrared light-emitting diodes, were secured rigidly to the anterior aspect of the L4 and L5 vertebral bodies using Kirschner wires to track motion using a motion analysis system (Optotrak Certus System; Northern Digital Inc., Waterloo, Canada). The locations of the markers, which denoted a rigid body, were aligned sagittal along the curvature of the spine. The TLIF model of L4–L5 segment was prepared according to the method described after the intact condition test [17]. The facet joints on right side were removed. Preparation of the disc space included removal of all disc material and endplate cartilage while preserving the lateral and anterior annulus. During preparation and test, the specimens were kept moist with 0.9% saline solution to prevent dehydration. All tests were carried out at room temperature, 27°C.

### Flexibility testing

Flexibility testing was performed in a similar way to the previously described methods [18]. The caudal end was fixed to the load frame of a custom-built six-degree-of-freedom spine simulator and a pure moment was applied to the L3 vertebra through servomotors. The direction of the load was aligned to induce flexion, extension, left and right lateral bending, and left and right axial rotation. Before recording motion data for each loading scenario, two preconditioning cycles were applied to the specimen to overcome the spine’s viscoelastic effects. There was no compressive preload applied on the specimens [19, 20]. A load control protocol was used to apply a maximum moment of ±5 Nm at a rate of 1°/sec [18]. The intervertebral rotation was obtained from the Optotrak Certus data files in the form of Euler angles (°) for the range of motion (ROM).

### Strain Measurement

In this study, the strain distribution on the vertebral surface was investigated by monoaxial strain gauges. Along with flexion and lateral bending motion configurations, strain distribution of the intact state and the fixation state was explored. Two 2.5 × 2.5 mm monoaxial strain gauges (TEST 3825; TEST ELECTRON Electronic Equipment Manufacturing Co., Ltd., Jiangsu, China) were spaced bilaterally on the lateral surface of the L4 and L5 vertebra at mid-height. Strain gauges were bonded with B-711 glue (TEST ELECTRON Electronic Equipment Manufacturing Co., Ltd., China) following an established procedure for wet cadaveric specimens [21–22]. The longitudinal axis of the strain gauge was maintained in parallel position with the longitudinal axis of the vertebra. The strains were sampled at 5,000 Hz using a multi-channel data-logger (TEST 3827 Strain Gauge Monitor; TEST ELECTRON Electronic Equipment Manufacturing Co., Ltd., China) with the signals transmitted from the testing machine and all other transducers. The principal tensile strains or compressive strains were computed on the basis of the readout from each strain gauge. Each loading configuration was repeated three times for each specimen, and the data of the third cycle was recorded.

### Study design

Each of the spines was initially tested in the intact state. After intact testing, all specimens underwent a righted sided TLIF at L4–L5 using a radiolucent interbody spacer. Two different test sequences were obtained for the randomized principle and structure damage control consideration. One sequence is unilateral pedicle screw and rod (UR); unilateral pedicle screw and plate (UP); UP+ transfacet pedicle screw (TFS); UR+TFS; UP+UR. The other sequence is UP; UR; UR+TFS; UP+TFS; UP+UR. Each specimen was then randomized tested by one of the sequence (Table 1). In UR+UP fixation mode, UP was used on the right side and UR was used on the left side. The TFS constructs always followed the unilateral pedicle screw constructs to avoid destroying the integrity of the facet joint and interfering the result of unilateral fixation. Because the pedicle screws used in plate system and rod system were different, it should be changed with respect to plate or rod fixation modes. To prevent loose of the screw pathway interface after repeated insertion and pullout of pedicle screws, 0.5 ml cement was injected into the screw pathway before setting screws.

**Table 1:** The testing order of seven calf spine specimens after intact test.

	First	Second	Third	Fourth	Fifth
1	UP	UR	UR+TFS	UP+TFS	UP+UR
2	UP	UR	UR+TFS	UP+TFS	UP+UR
3	UR	UP	UR+TFS	UP+TFS	UP+UR
4	UR	UP	UR+TFS	UP+TFS	UP+UR
5	UP	UR	UR+TFS	UP+TFS	UP+UR
6	UP	UR	UR+TFS	UP+TFS	UP+UR
7	UR	UP	UR+TFS	UP+TFS	UP+UR

UR, unilateral pedicle screw and rod; UP, unilateral pedicle screw and plate; UR+TFS, unilateral pedicle screw and rod + transfacet pedicle screw; UP+TFS, unilateral pedicle screw and plate + transfacet pedicle screw; UP+UR, unilateral pedicle screw and plate + unilateral pedicle screw and rod.

The polyaxial pedicle screw and rod system and monoaxial pedicle screw and plate system (Shanghai Sanyou Medical Instrument Co., Ltd., China) were used in the experiment. The diameters and lengths of the screws were 5.5 mm and 35 mm, respectively; the screw pitches sizes were 2.75mm on front half of screw shaft and 1.375mm on the rest; the diameter of the rod was 5.5 mm; the rod length was 35–40 mm; and the plate length was 40 mm. The TFS used was a modified pedicle screw (4.5 mm in diameter and 35 mm in length). Radiolucent PEEK cages with 5mm to 8 mm in height were made by Shanghai Sanyou Medical Instrument Company, which were lower than commonly used in human surgery and no radio-markers were made inside. We used fluoroscopy to confirm the right position of the pedicle screw, which was inserted parallel to the vertebral endplate (Fig 2).

### Data analysis

For all the constructs, both ROM and strain data were measured. Larger value of ROM indicated greater instability at index segment. A comparison of ROM data was performed using one-way repeated-measure analysis of variance followed by least significant difference post hoc analysis for multiple comparison procedures. Peirce criterion was applied to exclude outliers of the strain data [23]. Accordingly, 7.44% of the data had to be excluded (mainly due to strain gauge dysfunction) and none of the specimens had to be excluded. To avoid fictitiously increasing the coefficient of variation with close to zero data, only the strains higher than 50 microstrain were included [21]. To assess the significance of difference between strain values, nonparametric tests were performed because of the limited sample size: the significance of the difference between strain measurement locations was assessed separately for the different fixation configurations (Wilcoxon signed ranks test); the significance of the difference between the different fixation configurations was assessed separately for each strain measurement locations (Friedman Test). All statistical analyses were processed using SPSS for Windows, version 13.0 (SPSS, Chicago, IL, USA). A p value < 0.05 was considered significant.

## Results

### Kinematic results

#### Flexion-extension

**Fig 3:**
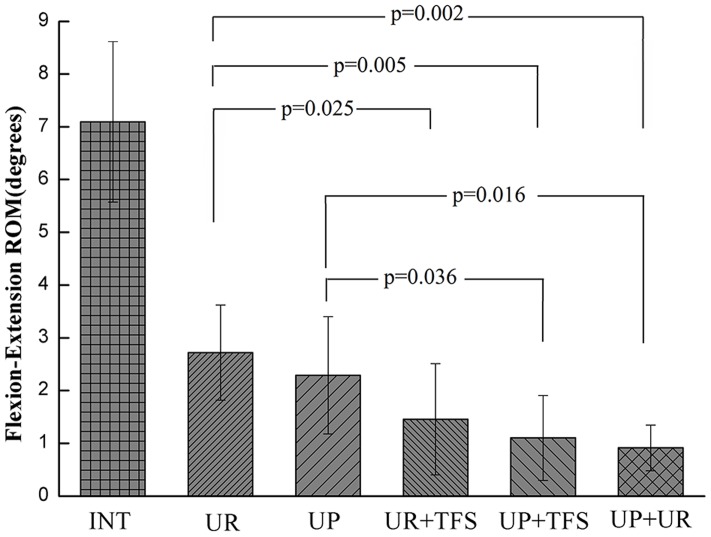
Flexion-extension ROM results. All comparisons related to intact case were significant (p<0.0001).

All instrumented constructs (UR, UP, UR+TFS, UP+TFS, UP+UR) significantly reduced ROM compared with the intact spine (7.1°±1.5°, p<0.001, Fig 3, Table 2). UP (2.3°±1.1°)reduced ROM significantly less than UP+TFS (1.1°±0.8°, p = 0.036)and UR (2.7°±0.9°)reduced ROM significantly less than UR+TFS (1.5°±1.0°, p = 0.025) and UP+TFS, while the difference was not significant between UR+TFS and UP constructs (p = 0.13). UP+UR, UP+TFS and UR+TFS constructs reduced ROM by 87%, 84% and 78%, respectively, and the difference between any of the these bilateral fixation instrumented constructs was not significant (p>0.338).

**Table 2:** L4-5 ROM (°) during different loading modes.

Motion	Intact	UR	UP	UR+TFS	UP+TFS	UR+UP
Flexion-extension	7.2±1.5	2.7±0.9	2.3±1.1	1.5±1.0	1.1±0.8	0.9±0.4
Lateral bending	9.7±2.0	3.6±0.7	3.4±1.0	2.1±1.0	1.7±0.9	0.6±0.4
Axial rotation	2.7±0.9	2.3±0.6	1.8±0.5	1.8±0.7	1.4±0.4	1.2±0.6

#### Lateral bending

**Fig 4:**
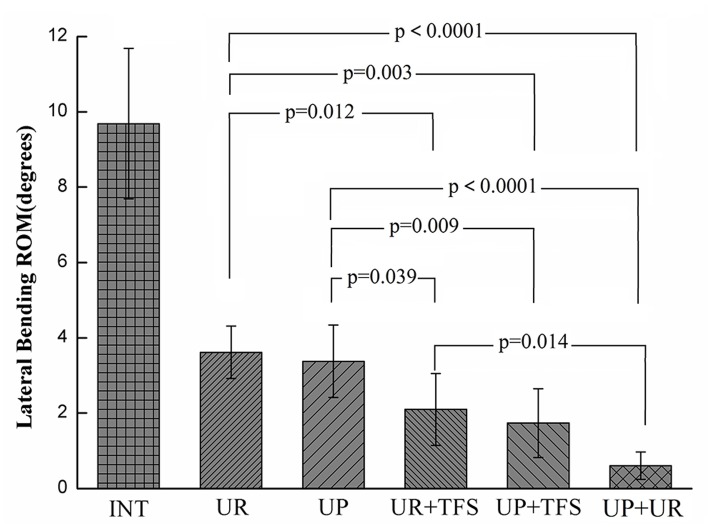
Lateral bending ROM results. All comparisons related to intact case were significant (p<0.0001).

UR (3.6°±0.7°), UP (3.4°±1.0°), UR+TFS (2.1°±1.0°), UP+TFS (1.7°±0.9°), and UP+UR (0.6°±0.4°) constructs significantly reduced ROM compared with the intact spine (9.7°±2.0°, p<0.001, Fig 4), by 63%, 65%, 78%, 83%, and 94%, respectively. The stability offered by UP+UR was significantly different from the other constructs (p<0.014), except the UP+TFS construct (p = 0.056). UR+TFS and UP+TFS reduced ROM significantly more than UR (p<0.012) and UP (p<0.039) constructs. The difference was not significant between UP and UR (p = 0.629) or UP+TFS and UR+TFS (p = 0.546).

#### Axial rotation

**Fig 5:**
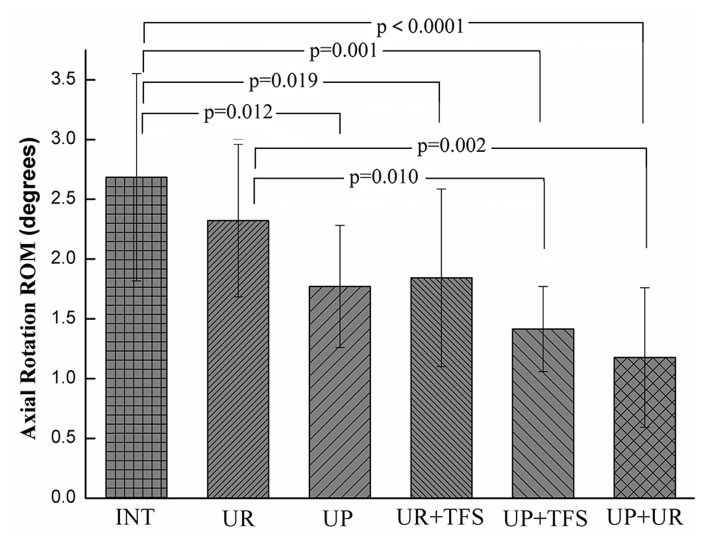
Axial rotation ROM results. Except UR fixation, all the other comparisons related to intact case were significant (p<0.012).

Only UR (2.3°±0.6°) construct did not significantly reduce ROM compared with the intact spine (2.7°±0.9°, p = 0.325), and all the other instrumented constructs demonstrated significant differences in stability (Fig 5). Furthermore, the UP (1.8°±0.5°) construct significantly reduced ROM compared with the intact spine (p = 0.012) and it even provided the equivalent stability to the UR+TFS (1.8°±0.7°) construct. ROM of UR construct was significantly less than the UP+TFS (1.4°±0.4°, p = 0.01) and UP+UR (1.2°±0.6°, p = 0.002) constructs, while the difference between UP and UP+TFS (p = 0.306) or UP+UR (p = 0.089) was not significant.

### Strain Distribution

**Fig 6:**
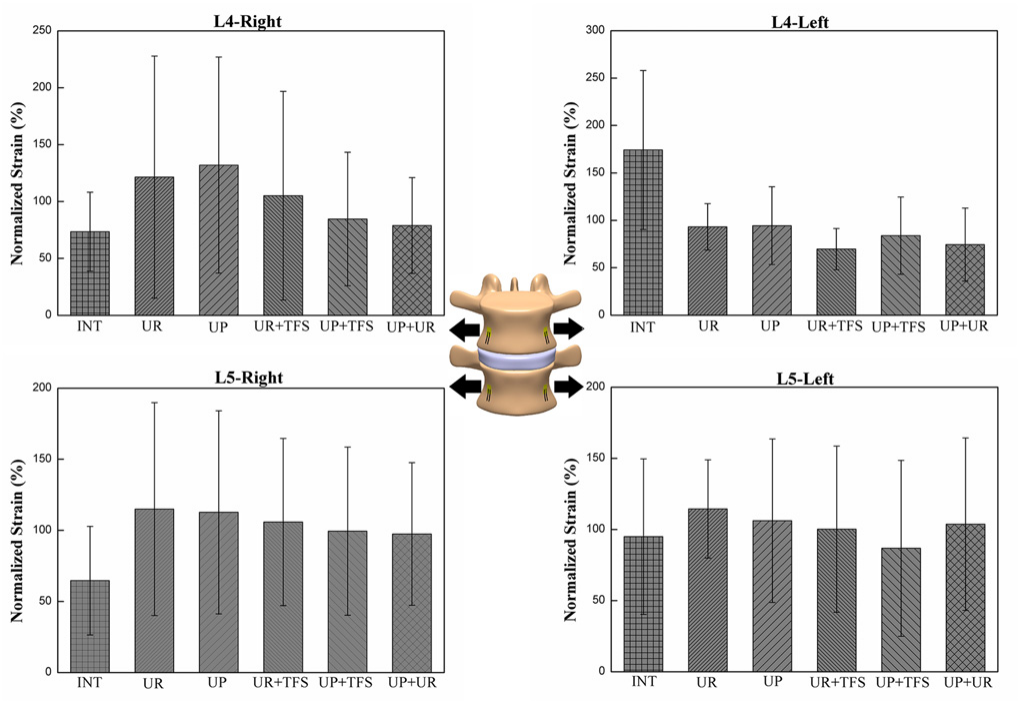
The compressive strains on the surface of the vertebral body for the different fixation modes under flexion motion. To enable comparisons among the different modes for each strain gauge, each strain was normalized with respect to the average among the fixation modes. The average and standard deviation of the seven specimens were plotted.

**Fig 7:**
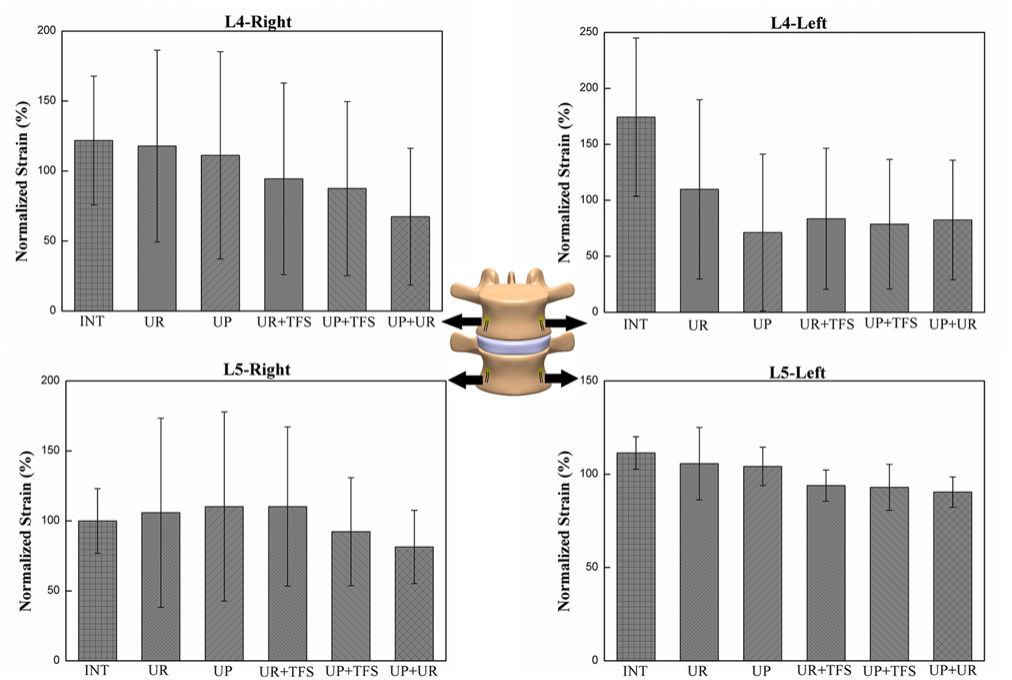
The compressive strains on the surface of the vertebral body for the different fixation modes under lateral bending motion. To make comparisons among the different modes for each strain gauge, each strain was normalized with respect to the average among the fixation modes. The average and standard deviation of the seven specimens are plotted.

The effect of fixation patterns in the same strain gauge with flexion and lateral bending motion configurations was presented in Figs 6 and 7. The strain pattern under flexion was compressive strain. Strain distribution on the left side (L4-L/L5-L) during left lateral bending and strain on the right side (L4-R/L5-R) during right lateral bending showed compressive strain. The strain distribution was significantly different between the left and right side of L4 vertebrae in UR (p = 0.043) in flexion and UR+TFS (p = 0.046) in lateral bending (Table 3). Statistically significant differences of strain distribution existed in L4-L, L4-R and L5-R strain gauges (p<0.010) in flexion and L4-L, L4-R and L5-L in lateral bending (p<0.015) (Table 4). This result indicated that strain distribution under different fixation states was different among these locations.

**Table 3:** Significance of the difference between left and right strain measurement location in L4 and L5 vertebrae under flexion and lateral bending motion for the different fixation configurations.

	L4		L5	
	Flexion	Lateral bending	Flexion	Lateral bending
INT	0.463	0.753	0.753	0.499
UR	0.043	0.116	0.345	0.735
UP	0.116	0.075	0.345	0.866
UR+TFS	0.08	0.046	0.116	0.499
UP+TFS	0.138	0.173	0.176	0.866
UP+UR	0.144	0.917	0.225	0.753

**Table 4:** Significance of the difference between the different fixation configurations in the four strain measurement locations.

Motion mode	L4-L	L4-R	L5-L	L5-R
Flexion	0.010	0.002	0.758	0.002
Lateral bending	0.015	0.001	0.010	0.085

## Discussion

In this study, calf spines were used, which were reported as the ideal alternative of human spines in biomechanical testing [24, 25]. However, there are some anatomical differences between human pedicle and calf pedicle [26, 27]. The ratio was about 0.8 in width and 1.5 in height of the coronal plane at the middle part of pedicle between human and 6–8 weeks calf pedicle. We measured the anatomic data in pretest and chose the proper size of pedicle screw. Therefore, we used smaller pedicle screws for fear of breaking the cortical bone of pedicle. The diameter of screw used in this study was 5.5 mm, while screw with 6.0 or 6.5 mm diameter was often used in human lumbar posterior instrumentation. This design reduces the comparable effect of this study. At the same time, to avoid the bias from specimens, different fixation statuses were performed on the same specimen. However, this protocol will cause new bias from the loose of bone-screw interface after repeated pullout and insertion of pedicle screws. So, we did pathway augmentation with cement before screw insertion to reduce the loose of bone-screw interface. All the testing was performed under pure moment loading without any compressive load of follower type. The quality of the data is similar with and without the application of a compressive preload. Hence, for the scope of this study, which is comparative evaluation of different instrumentation constructs, only pure moment was applied [19, 20]. In addition, the strain measurement value was derived from the third test value, because it may be greatly influenced by the creep effect of the specimen in the first circle. Furthermore, another reason was that strain value should be matched with ROM value, which was also picked up in the third cycle in this study.

Generally speaking, bilateral pedicle screw fixation provides more stabilization compared with the unilateral pedicle screws. The UP+UR fixation pattern is the most stable, which reduces the ROM to 12.5%, 6.2% and 44.4% in flexion-extension, lateral bending and axial rotation respectively, similar to those data reported by fixation with bilateral pedicle screw and rod pattern [9–11]. In lateral bending and flexion-extension, the application of contralateral TFS significantly increased the stability of fusion segment using either plate or rod system. The ROM reduced by UR construct is significantly less compared with UR+TFS construct and that reduced by UP construct is significant less compared with the UP+TFS construct. However, in axial rotation, the ROM reduced by UR construct is significantly less compared with the UP+TFS construct but not the UR+TFS construct. The ROM reduced by UP+TFS and UR+TFS is not significantly more compared with the UP construct, and even that reduced by UR+TFS construct is less than UP construct. This result indicates that the rod system has inherent deficiency in rotation stability while the plate system gets obvious improvement.

For the unilateral fixation, ROM of the fixation segment with UR construct is significantly less than that of the intact in flexion-extension and lateral bending, while the difference between UR construct and intact status is not significant in axial rotation. This result is consistent with other studies, which raises the concern of the insufficient stability offered by the UR fixation [8–10]. The ROM reduced by UP construct is14.8%, 5.6%, 21.7% more compared with the UR construct in flexion-extension, lateral bending and axial rotation. More importantly, there is significant difference between UP construct and intact status in axial rotation, which shows that the unilateral plate construct could make up the insufficient stability of unilateral rod construct. For the bilateral fixation, The ROM reduced by UP+TFS construct is 26.7%, 19.0%, 22.2% more compared with the UR+TFS construct in flexion-extension, lateral bending and axial rotation. These comparison results indicates that the plate systems superior to the rod system in ROM, especially in axial rotation of the TLIF model.

This study aims at assessing the effects of different fixation statuses on the strain distribution in lumbar vertebral body. The strain distribution was measured in the vertebral body of L4 and L5, under flexion and lateral bending motion configuration. The strain distribution was significantly affected by the loading modes in L4. Furthermore, the strain distribution was significantly different in UR in flexion (p = 0.043) and UR+TFS (p = 0.046) in lateral bending between the left and right side of L4 vertebra. This finding indicated that rod system could cause strain off-axis distribution and scoliosis potentially after fixation. In plate fixation status (UP, UP+TFS and UP+UR), the strain distribution on the left and right side was not significantly different, which indicated that plate system might offer better load transduction than the rod system. The difference could be partially explained by the different responses of the intervertebral discs to compression (a rather uniform pressure over the entire end plate) and traction (the nucleus pulposus has limited response to traction and most of the tensile force transferred by the annulus fibrosus).

In general, the results of the present study are consistent with other biomechanical studies in terms of asymmetrical posterior pedicle screw fixation reported in literature [8–10]. The UR construct was the least stable and the UR+TFS was close to bilateral pedicle screw and rod construct without significant difference in ROM test [8–10]. In the study by Goel et al. [28], unilateral and bilateral pedicle screw and plate constructs were compared in kinematic and finite-element methods. The result revealed that the unilateral plate was less rigid and was likely to reduce stress shielding of the vertebral bodies compared with the bilateral plate fixation model. The unilateral plate system presented coupled motions due to the inherent asymmetry and the likely inability to provide enough rigidity in posterior lateral fusion without intervertebral support. Another biomechanical study about the plate system by Crawford et al. [11] tested a new lumbar low-profile locking screw-plate construct. The conclusion was that posterior fixation using the plate system at L5–S1 offered stability, resistance to fatigue, and resistance to failure equivalent to fixation using a standard cantilevered pedicle screw-rod system. In this study, the plate system offered more stability compared with the rod system in unilateral fixation or unilateral fixation plus contralateral transfacet screw fixation for TLIF model.

## Conclusions

The biomechanical study indicates that the application of contralateral TFS construct provides significantly more stability than the unilateral fixation and provides similar stability with bilateral fixation. The result shows that the plate system offers more stability compared with the rod system, especially in axial rotation. The UP construct tested in the present study is biomechanically more stable compared with the UR construct, which could reduce the concern about the insufficient stability of the unilateral pedicle screw fixation. It needs further clinical studies to confirm whether UP fixation could achieve comparable clinical effect with UR fixation in MI-TLIF.
